# The role of inflammation in longitudinal renal function decline and incident chronic kidney disease: the multi-ethnic study of atherosclerosis

**DOI:** 10.1093/eurjpc/zwaf779

**Published:** 2025-12-23

**Authors:** Olayinka J Agboola, Jeffrey S K Chan, Jared A Spitz, Raymond N C Chan, Jaideep Patel, Charles German, Khurram Nasir, Michael J Blaha, Anurag Mehta, Garima V Sharma, Michael D Shapiro

**Affiliations:** Inova Schar Heart and Vascular, Inova Health System, Falls Church, VA, USA; Cardiovascular Analytics Group, China-UK Collaboration, Hong Kong, China; Norwich Medical School, University of East Anglia, Norwich NR4 7TJ, United Kingdom; School of Nursing and Health Studies, Hong Kong Metropolitan University, Kowloon, Hong Kong, China; Inova Schar Heart and Vascular, Inova Health System, Falls Church, VA, USA; Department of Medicine and Therapeutics, Prince of Wales Hospital, New Territories, Hong Kong, China; School of Biomedical Sciences, Faculty of Medicine, the Chinese University of Hong Kong, Hong Kong, China; Ciccarone Center for the Prevention of Cardiovascular Disease, Johns Hopkins School of Medicine, Baltimore, MD, USA; Section of Cardiology, Medstar Georgetown University Hospital, Washington, DC, USA; Section of Cardiology, Houston Methodist, Houston, TX, USA; School of Biomedical Sciences, Faculty of Medicine, the Chinese University of Hong Kong, Hong Kong, China; VCU Health Pauley Heart Center, Virginia Commonwealth University School of Medicine, Richmond, VA, USA; Inova Schar Heart and Vascular, Inova Health System, Falls Church, VA, USA; Center for Prevention of Cardiovascular Disease, Department of Cardiology, Wake Forest University School of Medicine, Bowman Gray Center for Medical Education, 475 Vine Street, Winston-Salem, NC 27101, USA

**Keywords:** chronic kidney disease, CKM syndrome, Inflammation, Interleukin-6, C-reactive protein

## Abstract

**Aims:**

Chronic inflammation has been implicated in renal decline, but long-term population-based data are limited. We aimed to evaluate the associations between baseline inflammatory markers [interleukin-6 (IL-6) and high-sensitivity C-reactive protein (hsCRP)] and long-term renal function decline and incident chronic kidney disease (CKD).

**Methods and results:**

This prospective cohort study included participants of the Multi-Ethnic Study of Atherosclerosis (MESA) with estimated glomerular function (eGFR) > 60 mL/min/1.73 m^2^ and available IL-6 and hsCRP measurements at visit 1. Outcomes included longitudinal changes in eGFR and log-transformed urinary albumin–creatinine ratio [log(UACR)] through visit 5, and incident CKD through 2018. Mixed-effect linear regression models assessed renal function decline. Fine-Gray regression was used to model incident CKD, accounting for competing mortality. Among 4680 (hsCRP) and 4580 (IL-6) participants (median follow-up for incident CKD: 16.8 years), higher IL-6 was consistently associated with faster eGFR decline [tertile 3 (T3; vs. T1): −0.32 mL/min/1.73m^2^/year (95% CI: 0.23–0.42 mL/min/1.73m^2^/year), *P* < 0.001] greater log[UACR] increase [T3: 6.67 × 10^−3^ unit/year (2.95 × 10–3–10.39 × 10^−3^ unit/year), *P* < 0.001], and higher CKD incidence [T3: sub-hazard ratio 1.65 (1.23–2.20), *P* = 0.001]. Contrastingly, hsCRP exhibited weaker and inconsistent associations.

**Conclusion:**

Elevated IL-6 predicts greater long-term eGFR decline, worsening albuminuria, and higher CKD risk. These results highlight IL-6’s potential as a renal risk biomarker, meriting further study in cardiovascular–kidney–metabolic (CKM) syndromes.

**Lay summary:**

This study investigated how inflammation is related to deteriorations in kidney function and the occurrence of chronic (long-term) kidney disease (CKD), both of which are critical components of the cardiovascular–kidney–metabolic (CKM) syndrome. Using data from the Multi-Ethnic Study of Atherosclerosis, over 4500 volunteers had inflammation markers measured and were followed for almost 17 years. We found that higher levels of inflammation were correlated with worse deterioration in kidney function and higher risks of developing CKD. These findings reaffirmed the pivotal role of inflammation in the long-term development of kidney disease. This study highlights the need for further investigation of how inflammatory markers can be used to predict future heart and kidney disease, especially as new drugs to treat inflammation are being developed.

## Graphical Abstract

**Graphical Abstract:**
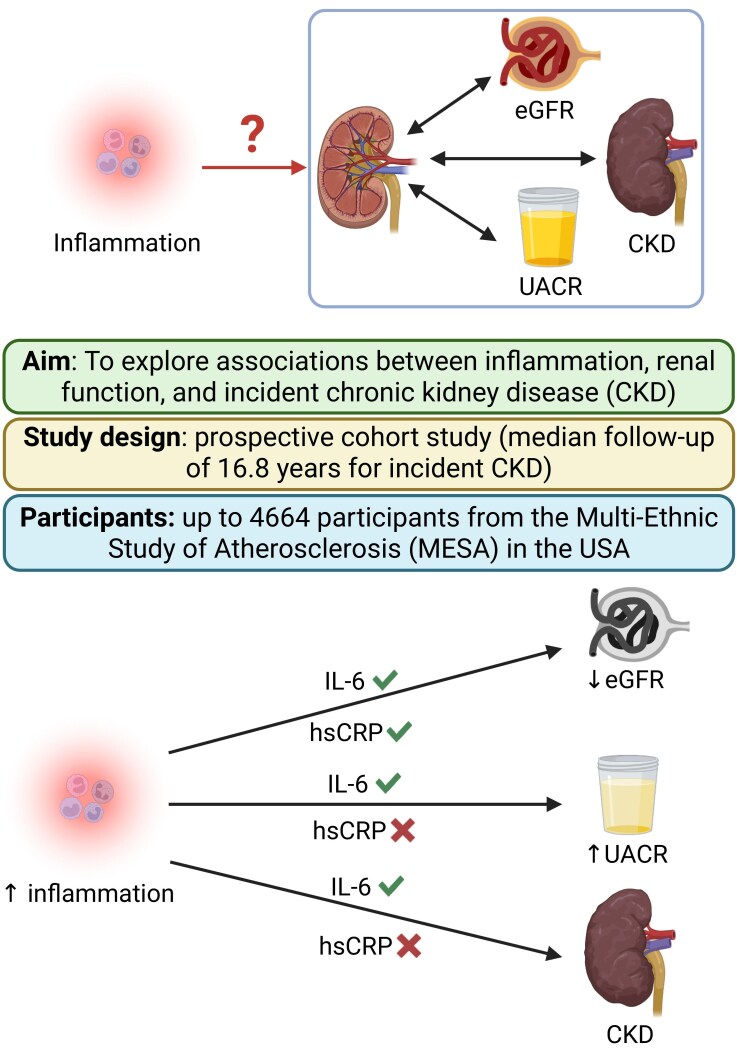

**See the editorial comment for this article ‘Interleukin-6 and the inflammatory origins of chronic kidney disease’, by A. Chang**  ***et al*****., https://doi.org/10.1093/eurjpc/zwag088.**

## Introduction

Chronic kidney disease (CKD) and cardiovascular disease (CVD) frequently coexist within the broader cardiovascular–kidney–metabolic (CKM) syndrome, a framework that highlights their interrelated pathophysiology and shared risk factors.^1^ CKM syndrome is driven by excess or dysfunctional adipose tissue, which promotes a proinflammatory and prooxidative state. This inflammatory milieu accelerates metabolic derangements, atherosclerosis, and progressive renal injury, ultimately resulting in multiorgan damage, including overt CVD, stroke, heart failure, and kidney failure.^1^

Beyond traditional metabolic risk factors, inflammation has emerged as a critical driver of CKM progression. In atherosclerosis, inflammatory pathways, including immune cell activation, cytokine release, and endothelial dysfunction, underlie plaque formation, progression, and rupture.^2,3^ A component of the residual risk hypothesis is that despite optimal lowering of low-density lipoprotein cholesterol, persistent low-grade inflammation continues to contribute to cardiovascular events.^2,4,5^ Interleukin-6 (IL-6), an upstream cytokine in this cascade, has been shown to stimulate hepatic synthesis of C-reactive protein (CRP), the classic downstream inflammatory marker used in clinical practice.^6,7^ While high-sensitivity CRP (hsCRP) is widely measured, recent evidence indicates that IL-6 may be a more robust and consistent predictor of adverse cardiovascular outcomes, given its more direct reflection of upstream immune activity.^6,7^

Parallel to its role in atherosclerosis, inflammation is also implicated in the initiation and progression of CKD.^8,9^ Elevated IL-6 levels have been linked with renal fibrosis, glomerular and tubular injury, and proteinuria through pathways such as the NLRP3 inflammasome and Janus kinase (JAK)/STAT3 signaling.^10,11^ However, much of the evidence connecting IL-6 with renal function decline has been limited to cross-sectional analyses, short follow-up, or single ethnic cohorts.^9,12,13^ In the Multi-Ethnic Study of Atherosclerosis (MESA), some studies have demonstrated associations between inflammatory markers and kidney function decline, but long-term data remain sparse.^13,14^

Therefore, we aimed to evaluate the relationship between baseline IL-6 and hsCRP levels and long-term changes in estimated glomerular filtration rate (eGFR), albuminuria progression, and incident CKD within the MESA cohort. We hypothesized that higher IL-6 and hsCRP levels would be associated with greater renal decline and higher CKD incidence.

## Methods

### Source of data

The Multi-Ethnic Study of Atherosclerosis is an NHLBI-funded population-based cohort study designed to understand the characteristics of subclinical cardiovascular disease and the risk factors that predict the progression of subclinical cardiovascular disease to overt disease. The study design has been previously described.^15,16^ In summary, 6814 men and women aged 45–84 from four different ethnic groups (white—38%, African American—28%, Hispanic—22%, and Chinese—12%) were drawn from six field centres across the USA. The first examination of participants took place from July 2000 to July 2002. There have been six follow-up examinations after the first examination. MESA was approved by each of the institutional review boards at the participating centres, and all participants provided written informed consent.

### Exposures, outcomes, and follow-up

The exposures of interest were baseline levels of IL-6 and hsCRP measured during visit 1, which were analysed in tertiles. Biomarkers were assayed on fasting plasma or serum stored at −70°C. The hsCRP concentration was measured using a BNII nephelometer (high-sensitivity CRP; Dade Behring Inc.), with intra-assay coefficients of variation (CV) ranging 2.3–4.4% and inter-assay CVs of 2.1–5.7%. IL-6 was measured using an ultrasensitive enzyme-linked immunosorbent assay (Quantikine HS Human IL-6 Immunoassay; R&D Systems, www.rndsystems.com), with an analytical CV of 6.3%.^16^

The outcomes were longitudinal changes in eGFR and urine albumin-to-creatinine ratio (UACR), as well as incident CKD. Longitudinal changes in eGFR and UACR were analysed as continuous variables with repeated measures, with UACR log-transformed as log[UACR].

Repeated eGFR and UACR measures were available through visit 5. Follow-up was limited to visit 5 to reduce bias from differential loss due to illness or death. Incident CKD was defined at the first diagnosis after baseline and analysed through the end of 2018, with all-cause mortality treated as a competing event.

### Inclusion and exclusion criteria

All participants of MESA at visit 1 were eligible for inclusion. All participants with an eGFR of <60 mL/min/1.73m^2^ at visit 1 were excluded. Participants with missing baseline eGFR or covariate data (see ‘*Covariates*’) were excluded from all analyses, and those with missing IL-6 and hsCRP measurements at visit 1 were excluded from the respective analyses.

For analyses of longitudinal changes in eGFR, participants without any follow-up eGFR measurement, and those without any complete set of eGFR and covariate data (see ‘*Covariates*’) for any follow-up visit (i.e. after visit 1) were additionally excluded. For analyses of longitudinal changes in UACR, participants without any follow-up UACR measurement, and those without any complete set of UACR and covariate data (see ‘*Covariates*’) for any follow-up visit were additionally excluded. For analyses of incident CKD, participants with missing follow-up data were additionally excluded.

### Definition and measurements of estimated glomerular function and urinary albumin–creatinine ratio

Serum creatinine was measured using standardized spectrophotometric methods, and eGFR was calculated from the serum creatinine level, sex, and age using the 2021 CKD Epidemiology Collaboration (CKD-EPI) equation.^17^ UACR was measured using standardized nephelometric methods.

### Ascertainment of incident chronic kidney disease

Clinical events in MESA were collected from medical records, death certificates, autopsy reports, interviews from participants, and, in some instances, interviews or questionnaires from participants’ physicians, relatives, or friends. Periodic follow-up interviews were conducted every 6–12 months between examinations to ascertain clinical events.

### Covariates

Covariates included age, sex, race/ethnicity, education level, waist and hip circumference, body-mass index, lipid levels, fasting glucose, diabetes mellitus, systolic and diastolic blood pressure, hypertension, current alcohol use, smoking status, and use of relevant medications (angiotensin-converting enzyme inhibitors or angiotensin receptor blockers, other antihypertensives, statins, and fibrates). Body-mass index was calculated as $\frac{\mathrm{body}\;\mathrm{weight}\;(\mathrm{kg})}{{\left(\mathrm{body}\;\mathrm{height}\;[m]\right)}^{2}}$. Hypertension was defined per the sixth report of the Joint National Committee on Prevention Detection, Evaluation, and Treatment of High Blood Pressure, i.e. a systolic blood pressure of ≥140 mmHg or a diastolic blood pressure of ≥90 mmHg,^18^ and/or the use of antihypertensive medication(s). Diabetes mellitus was defined per the 2003 American Diabetes Association fasting criteria algorithm, i.e. a fasting glucose level of ≥126 mg/dL,^19^ and/or the use of insulin or oral hypoglycaemic agent(s). Smoking status (never, former, or current smoker) and current alcohol use were both self-reported. The Agatston score measured at exam 1 was also recorded, using standardized protocols which were published previously.^20^

### Statistical analysis

Continuous variables are summarized as medians with interquartile ranges, while categorical variables are summarized as counts and percentages. All *P*-values were two-sided, with *P* < 0.05 considered statistically significant. All analyses were performed using Stata v16.1 (StataCorp LLC, College Station, TX, USA).

Group differences were assessed using Kruskal–Wallis or χ^2^ tests. For repeated measures [eGFR and log(UACR)], multivariable two-level mixed-effect linear models were used, with measurements nested within individual participants. Random intercepts were allowed for the time of measurement (measured from visit 1 in years), exposure of interest (IL-6 or hsCRP), and covariates, and random slopes were allowed for the time of measurement. All variances and covariances were distinctly estimated alongside a robust variance estimator. All linear models were adjusted for the aforementioned covariates (see ‘*Covariates*’), which were pre-specified based on clinical and epidemiological knowledge. The interaction between the exposure variable and the time of measurement was used to compare the rate of change in the outcome between groups of exposure. In addition to the models presented in this manuscript, additional models were explored where the square-term and cubic-term of the time of measurement were allowed to interact with the exposure. However, the Akaike information criteria suggested that there were only minimal improvements in model fit, and likelihood-ratio tests indicated that these models did not result in significantly better model fit than the simpler models that only considered linear changes of the outcome with time. For this reason, the latter models were used and reported in this manuscript. Regression coefficients (*β*) and the corresponding 95% confidence intervals (CIs) were used as summary statistics. The regression coefficients for the eGFR analyses were interpreted as the difference in annual eGFR reduction compared to tertile 1, with the unit of mL/min/1.73m^2^ per year. Meanwhile, those for the log[UACR] analyses were interpreted as the difference in annual log[UACR] increase compared to tertile 1 – due to the magnitude of observed effect sizes, the regression coefficients for the log[UACR] analyses were expressed in 10^−3^ unit per year.

For incident CKD, multivariable Fine-Gray sub-distribution models were used, with all-cause mortality treated as a competing event. Competing events prevent the occurrence or observation of the outcome of interest. In the case of this study, it is impossible for incident CKD to occur after mortality, meaning that mortality can be seen as a competing event for incident CKD. The Fine-Gray sub-distribution model was chosen over the Cox proportional hazards model as the former can intrinsically account for competing events and generally provides less biased estimates of the cumulative incidence function. All models were adjusted for the aforementioned, pre-specified covariates at visit 1 (see ‘*Covariates*’). The proportional sub-hazard assumption was tested using interactions with the time-to-event variable, with no violation found. Sub-hazard ratios (SHRs) and the corresponding 95% CIs were used as summary statistics. Aalen–Johansen curves were used to visualize the cumulative incidence of incident CKD over time, stratified by tertiles of the exposures.

Four pre-specified subgroup analyses were performed for each outcome and exposure, stratifying by age (≤60 years old vs. >60 years old; cut-off chosen for clinical relevance and ease of interpretation), baseline eGFR (<90 mL/min/1.73m^2^ vs. ≥90 mL/min/1.73m^2^; cut-off chosen for clinical relevance), the 10-year atherosclerotic cardiovascular disease (ASCVD) risk as estimated by the base PREVENT model (<5% vs. ≥5%; cut-off was chosen in view of the threshold for preventive statin prescriptions recommended by contemporary guidelines, which was based on older cardiovascular risk stratification tools), and baseline glycaemic dysregulation (defined as the presence of impaired fasting glucose or diabetes mellitus). Five *post hoc* subgroup analyses were performed as well for each outcome and exposure, stratifying by sex (male vs. female), obesity (per the definition in American Heart Association’s definition of CKM stage 1, i.e. BMI ≥25 kg/m2 (or ≥23 kg/m2 if Asian ancestry), or waist circumference ≥88/102 cm in women/men (or, if Asian ancestry, ≥80/90 cm in women/men),^1^) ethnicity (white vs. non-white), and binarily categorized coronary calcification (Agatston score of 0 vs. >0). To ensure robustness of the definition of obesity used and categorization of Agatston score, respectively, additional subgroup analyses were performed with stratification by BMI-defined obesity (i.e. the BMI component of the aforementioned definition of obesity), abdominal obesity (i.e. the waist circumference component of the aforementioned definition of obesity), and ordinally categorized coronary calcification (Agatston score of 0 vs. >0−<100 vs. ≥100). Subgroup interactions were tested jointly using a χ^2^-based test.

Two sensitivity analyses were performed. In the first, pre-specified sensitivity analysis, the main analyses for all outcomes were repeated with IL-6 and hsCRP analysed as continuous variables instead of tertiles—this allowed simpler and more direct comparison of the strength of association between the two exposures and each outcome, as well as further ensuring robustness of the tertile-based analyses. As the data distributions of both IL-6 and CRP were right-skewed, both variables were log-transformed (as log[IL-6] and log[hsCRP], respectively; there were no zeroes for both variables) and then standardized for this sensitivity analysis, such that all estimates represented effects per standard deviation (SD) increment in the log-transformed exposure. Additional models with the exposures modelled using three- and five-knot restricted cubic splines were explored, with knots placed at the conventional percentiles per recommendations by Harrell.^21^ However, as the Akaike information criterion indicated that these models did not result in a meaningfully better fit than the above model using standardized log-transformed exposure variables, and in view of the relatively difficult interpretation and reporting of these models, they are not presented in this manuscript. The lack of improvement in model fit also suggested that non-linearity in the associations between the exposures and the outcomes was unlikely to be important.

In the second, *post hoc* sensitivity analysis, two models were constructed based on the main analyses for all outcomes, with further adjustments for log-transformed and ordinally categorized Agatston score, respectively. This was conducted as the Agatston score is a marker of atherosclerotic burden, which, considering the definition and stages of CKM syndrome, may influence one’s renal health over time. Logarithmic transformation of the Agatston score was performed as the untransformed variable exhibited substantial right skew and was calculated as ln[(Agatston score) + 1] in order to accommodate Agatston scores of 0. This handling of the Agatston score is consistent with prior studies.^22–24^

Further *post hoc* sensitivity analyses were performed for incident CKD to ensure the findings’ robustness against reverse causation. In the third sensitivity analysis, two Fine-Gray sub-distribution models were constructed, in which participants who developed early events (i.e. within the first 3 and 5 years of follow-up, respectively) were censored at the date of event occurrence—this was done as the development of CKD occurs insidiously, meaning that early events are unlikely to be due to the effects of inflammation. In the fourth sensitivity analysis, participants who developed major adverse cardiovascular events (MACE) within 3 years of exam 1 without prior incident CKD were censored at the date of MACE occurrence. MACE was defined as the first occurrence of non-fatal myocardial infarction, non-fatal stroke, heart failure, or resuscitated cardiac arrest, all of which were ascertained by clinical records review by the MESA study committee. This last sensitivity analysis was done because, per the definition and stages of CKM syndrome, clinical cardiovascular events predispose to renal dysfunction and CKD.

## Results

**Figure 1:**
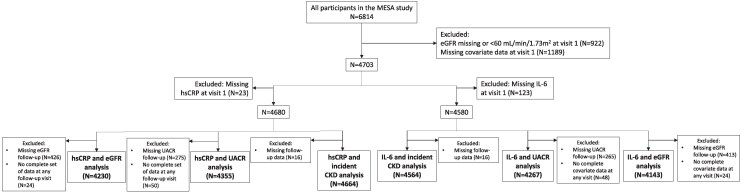
Study flow diagram. CKD, chronic kidney disease; eGFR, estimated glomerular filtration rate; hsCRP, high-sensitivity C-reactive protein; IL-6, interleukin-6; UACR, urinary albumin–creatinine ratio; and MESA, Multi-ethnic Study of Atherosclerosis.

Amongst all participants of the MESA study, 4680 had complete covariate data and a non-missing eGFR of ≥60 mL/min/1.73m^2^ at visit 1 with a non-missing CRP measurement at visit 1, and 4580 had complete covariate data and a non-missing eGFR of ≥60 mL/min/1.73m^2^ at visit 1 with a non-missing IL-6 measurement at visit 1. The respective numbers of participants included in the analysis of the association between each outcome and hsCRP and IL-6 are summarized in *Figure 1*. The baseline (i.e. visit 1) characteristics of participants included in the analysis of associations between longitudinal eGFR changes and IL-6 levels are summarized in *Table 1*. At baseline, participants with the highest tertile of IL-6 level were generally older, had more cardiometabolic comorbidities and risk factors (except current alcohol use), had worse UACR, and had higher 10-year ASCVD risk as estimated by the core PREVENT equation.

**Table 1:** Baseline characteristics of participants included in the analysis of associations between longitudinal estimated glomerular filtration rate changes and interleukin-6 levels, stratified by tertiles of interleukin-6 levels at visit 1

	Total cohort	Tertile 1	Tertile 2	Tertile 3	Between-tertile *P*-value
	*n* = 4143	*n* = 1381	*n* = 1381	*n* = 1381	
Age, years	59 [52–68]	56 [50–64]	60 [53–68]	62 [53–69]	<0.001
Female, *n* (%)	1949 (47.0)	584 (42.3)	650 (47.1)	715 (41.8)	<0.001
Race, *n* (%)					<0.001
White	1812 (43.7)	673 (48.7)	608 (44.0)	531 (38.5)	
Chinese	282 (6.8)	158 (11.4)	77 (5.6)	47 (3.4)	
Black	1188 (28.7)	311 (22.5)	413 (29.9)	464 (33.6)	
Hispanic/Latino	861 (20.8)	239 (17.3)	283 (20.5)	339 (24.6)	
Education, *n* (%)					<0.001
No schooling	25 (0.6)	6 (0.4)	6 (0.4)	13 (0.9)	
Grades 1–8	307 (7.4)	97 (7.0)	99 (7.2)	111 (8.0)	
Grades 9–11	239 (5.8)	52 (3.8)	80 (5.8)	107 (7.8)	
Completed high school	680 (16.4)	184 (13.3)	230 (16.7)	266 (19.3)	
Some college but no degree	696 (16.8)	222 (16.1)	223 (16.2)	251 (18.2)	
Technical school certificate	314 (7.6)	90 (6.5)	114 (8.3)	110 (8.0)	
Associate degree	218 (5.3)	66 (4.8)	80 (5.8)	72 (5.2)	
Bachelor’s degree	804 (19.4)	325 (23.5)	263 (19.0)	216 (15.6)	
Graduate or professional school	860 (20.8)	339 (24.6)	286 (20.7)	235 (17.0)	
Body-mass index, kg m^−2^	27.8 [24.7–31.2]	25.8 [23.2–28.4]	28.1 [25.1–31.2]	29.9 [26.5–34.5]	<0.001
Waist circumference, cm	98 [89–107]	92 [84–100]	98 [90–107]	103 [95–113]	<0.001
Hip circumference, cm	104 [99–112]	101 [96–106]	105 [99–112]	108 [101–118]	<0.001
Current alcohol use, *n* (%)	2897 (69.9)	1031 (74.7)	957 (69.3)	909 (65.8)	<0.001
Smoking history, *n* (%)					<0.001
Never	1780 (43.0)	659 (47.7)	594 (43.0)	527 (38.2)	
Former	1747 (42.2)	562 (40.7)	582 (42.1)	603 (43.7)	
Current	616 (14.9)	160 (11.6)	205 (14.8)	251 (18.2)	
hsCRP level, mg/L	1.83 [0.80–4.08]	0.89 [0.46–1.77]	1.90 [0.99–3.62]	3.95 [1.86–7.70]	<0.001
IL-6, pg/mL	1.14 [0.73–1.81]	0.62 [0.49–0.73]	1.14 [0.99–1.31]	2.26 [1.81–3.15]	<0.001
Total cholesterol, mg/dL	192 [170–214]	193 [172–216]	195 [173–215]	188 [166–211]	<0.001
Triglycerides, mg/dL	107 [75–155]	98 [70–141]	112 [78–161]	111 [80–160]	<0.001
HDL cholesterol, mg/dL	48 [40–59]	51 [42–63]	48 [40–59]	46 [39–56]	<0.001
LDL cholesterol, mg/dL	117 [96–136]	116 [98–137]	119 [98–138]	114 [94–134]	0.001
Diastolic blood pressure, mmHg	72 [65–79]	72 [65–78]	72 [66–79]	72 [65–79]	0.675
Systolic blood pressure, mmHg	121 [110–137]	117 [106–131]	122 [111–138]	126 [114–141]	<0.001
eGFR, mL/min/1.73m^2^	80 [72–91]	81 [72–91]	81 [72–91]	80 [71–90]	0.070
Urine albumin–creatinine ratio, mg/g	4.8 [3.1–9.1]	4.2 [2.9–7.0]	4.8 [3.1–9]	5.8 [3.5–12.4]	<0.001
Hypertension, *n* (%)	1617 (39.0)	378 (27.4)	565 (40.9)	674 (48.8)	<0.001
Diabetes mellitus, *n* (%)					<0.001
Normal	3177 (76.7)	1168 (84.6)	1055 (76.4)	954 (69.1)	
Impaired fasting glucose	532 (12.8)	126 (9.1)	184 (13.3)	222 (16.1)	
Untreated diabetes mellitus	92 (2.2)	19 (1.4)	30 (2.2)	43 (3.1)	
Treated diabetes mellitus	342 (8.3)	68 (4.9)	112 (8.1)	162 (11.7)	
ACEI/ARB, *n* (%)	636 (15.4)	148 (10.7)	208 (15.1)	280 (20.3)	<0.001
Statin, *n* (%)	574 (13.9)	182 (13.2)	205 (14.8)	187 (13.5)	0.412
Fibrates, *n* (%)	264 (1.2)	59 (0.8)	97 (1.4)	108 (1.6)	<0.001
Antihypertensive, *n* (%)	1339 (32.3)	324 (23.5)	457 (33.1)	558 (40.4)	<0.001
10-year risk of ASCVD as estimated using the base PREVENT equation, %	4.2 [2.2–8.2]	3.0 [1.7–5.7]	4.5 [2.4–8.5]	5.7 [2.7–9.8]	<0.001
Agatston score	0 [0–65.0]	0 [0–26.6]	0 [0–78.1]	3.31 [0–109.1]	<0.001
Agatston score categories, *n* (%)					<0.001
0	2217 (53.5)	848 (61.4)	722 (52.3)	647 (46.9)	
> 0−<100	1055 (25.5)	327 (23.7)	352 (25.5)	376 (27.2)	
≥ 100	871 (21.0)	206 (14.9)	307 (22.2)	358 (25.9)	

Continuous variables are summarized as medians with interquartile ranges in brackets.

ACEI, angiotensin-converting enzyme inhibitor; ARB, angiotensin receptor blocker; ASCVD, atherosclerotic cardiovascular disease; eGFR, estimated glomerular filtration rate; HDL, high-density cholesterol; hsCRP, high-sensitivity C-reactive protein; IL-6, interleukin-6; LDL, low-density cholesterol.

### Longitudinal estimated glomerular function changes

Among both cohorts for the analysis of associations between IL-6 (*n* = 4143) and hsCRP (*n* = 4230) and longitudinal eGFR changes, there were a median of 4 (interquartile range: 3–4) eGFR measurements across a median of 9.2 (interquartile range: 8.6–9.6) years and 9.2 (interquartile range: 8.5–9.6) years, respectively. The unadjusted estimated annual decline in eGFR, stratified by tertiles of IL-6 and hsCRP, is summarized in Supplementary material online, *Table S1*.

**Figure 2:**
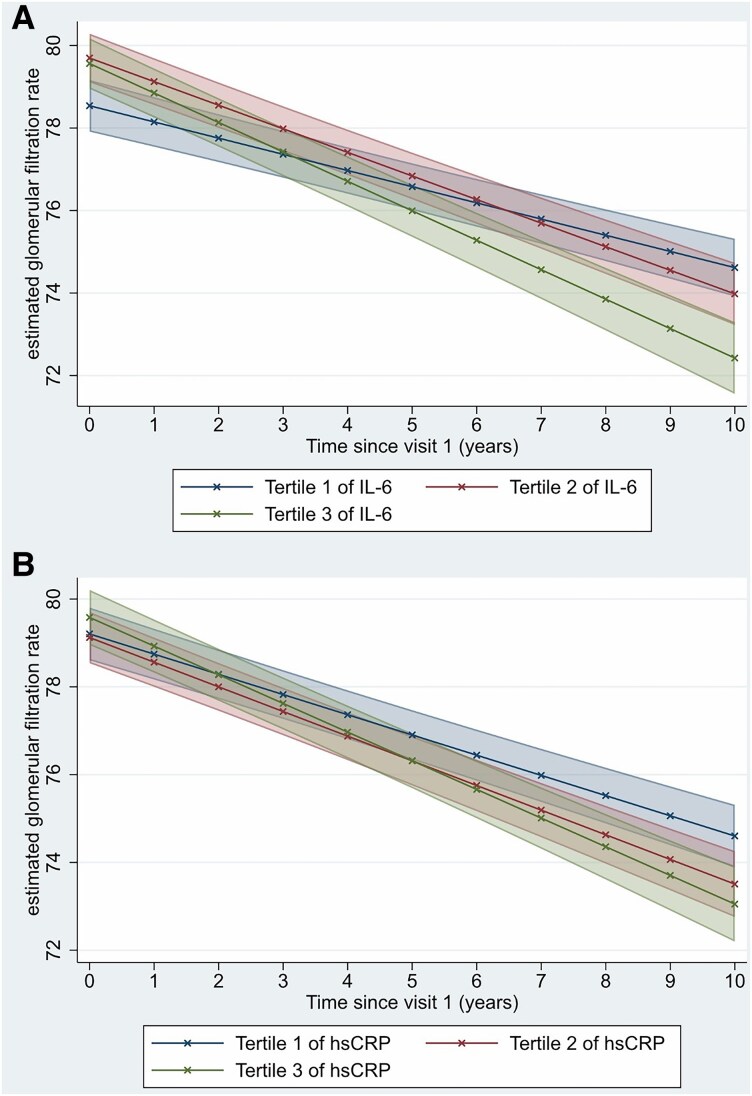
Adjusted estimated glomerular filtration rates (in mL/min/1.73m^2^) for each tertile of (*A*) interleukin-6 (IL-6) and (*B*) high-sensitivity C-reactive protein (hsCRP) over time.

Results of the main and subgroup analyses for longitudinal eGFR changes are summarized in *Table 2*. Compared to participants in tertile 1 of IL-6, participants in tertile 2 [*β* (in mL/min/1.73m^2^ per year): 0.18 (95% CI: 0.09–0.27), *P* < 0.001] and tertile 3 [β: 0.32 (95% CI: 0.23–0.42), *P* < 0.001] of IL-6 had significantly greater annual eGFR reduction (*Figure 2A*). Similarly, compared to participants in tertile 1 of hsCRP, participants in tertile 2 [*β*: 0.10 (95% CI: 0.02–0.19), *P* = 0.026] and tertile 3 [*β*: 0.19 (95% CI: 0.10–0.29), *P* < 0.001] of hsCRP had significantly greater annual eGFR reduction (*Figure 2B*). In the first sensitivity analysis (in which exposures were analysed as standardized log-transformed continuous variables), standardized log-transformed IL-6 was consistently associated with a significant annual decline in eGFR [β per SD-increment in log-transformed IL-6: 0.17 (95% CI: 0.13–0.20), *P* < 0.001], as was standardized log-transformed hsCRP [*β* per SD-increment in log-transformed hsCRP: 0.11 (95% CI: 0.08–0.15), *P* < 0.001]. In the second sensitivity analysis, further adjustments for the Agatston score yielded similar results as the main analyses (see Supplementary material online, *Table S2*).

**Table 2:** Summary of the main analytic results. Bracketed numbers represent 95% confidence intervals

Outcome	IL-6			hsCRP		
	Tertile 1	Tertile 2	Tertile 3	Tertile 1	Tertile 2	Tertile 3
Longitudinal changes in eGFR^a^ (IL-6: *n* = 4143; hsCRP: *n* = 4230)	0 (reference)	0.18 [0.09–0.27], *P* < 0.001	0.32 [0.23–0.42], *P* < 0.001	0 (reference)	0.10 [0.02–0.19], *P* = 0.026	0.19 [0.10–0.29], *P* < 0.001
Longitudinal changes in log[UACR]^b^ (IL-6: *n* = 4267; hsCRP: *n* = 4355)	0 (reference)	3.95 [0.46–7.43], *P* = 0.027	6.67 [2.95–10.39], *P* < 0.001	0 (reference)	5.08 [1.56–8.61], *P* = 0.005	2.96 [−0.74–6.66], *P* = 0.117
Incident chronic kidney disease^c^ (IL-6: *n* = 4564; hsCRP: *n* = 4664)	1 (reference)	1.43 [1.08–1.89], *P* = 0.013	1.65 [1.23–2.20], *P* = 0.001	1 (reference)	1.05 [0.82–1.34], *P* = 0.716	1.10 [0.85–1.44], *P* = 0.467

eGFR, estimated glomerular filtration rate; hsCRP, high-sensitivity C-reactive protein; IL-6, interleukin-6; SHR, sub-hazard ratio; UACR, urinary albumin–creatinine ratio.

^a^Estimates represent annual eGFR reduction compared to tertile 1, with the unit of mL/min/1.73m^2^ per year.

^b^Estimates represent differences in the annual log[UACR] increase compared to tertile 1, with the unit of 10^−3^ unit per year.

^c^Estimates are sub-hazard ratios.

Subgroup analyses for both IL-6 and hsCRP produced estimates that were directionally consistent in all subgroups (see Supplementary material online, *Table S2*). For IL-6, significantly stronger associations were observed in participants aged ≥61 years old (joint test for subgroup interaction *P* = 0.022), participants with ≥5% 10-year ASCVD risk (joint test for subgroup interaction *P* = 0.014), and participants with hypertension (joint test for subgroup interaction *P* = 0.048). No other statistically significant subgroup interactions were found.

### Longitudinal urinary albumin–creatinine ratio changes

Among both cohorts for the analysis of associations between IL-6 (*n* = 4267) and hsCRP (*n* = 4355) and longitudinal UACR changes, there were a median of 4 (interquartile range: 3–4) UACR measurements across a median of 9.2 (interquartile range: 3.7–9.6) years. The unadjusted estimated annual increase in log[UACR], stratified by tertiles of IL-6 and hsCRP, is summarized in Supplementary material online, *Table S3*.

**Figure 3:**
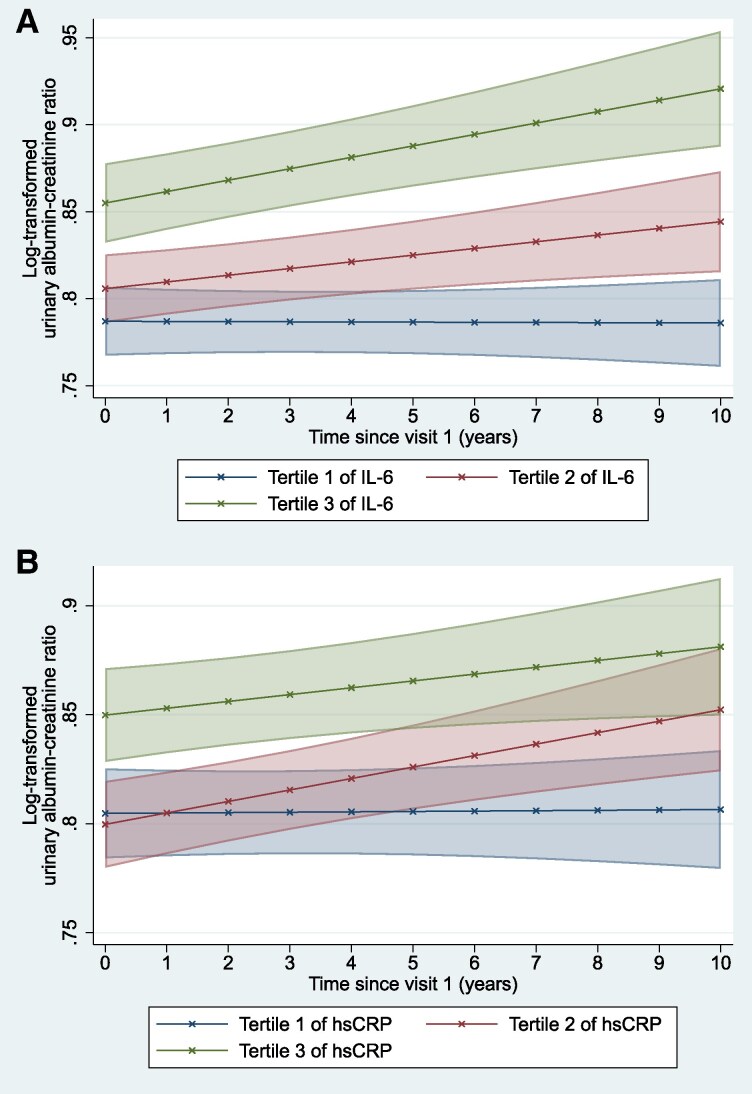
Adjusted log-transformed urinary albumin–creatinine ratio for each tertile of (*A*) interleukin-6 (IL-6) and (*B*) high-sensitivity C-reactive protein (hsCRP) over time.

Results of the main and subgroup analyses for longitudinal UACR changes are summarized in *Table 2*. Compared to participants in tertile 1 of IL-6, participants in tertile 2 (β [in ×10^−3^ unit per year]: 3.95 [95% CI: 0.46–7.43], *P* = 0.027) and tertile 3 [β: 6.67 (95% CI: 2.95–10.39), *P* < 0.001] of IL-6 had significantly greater annual increase in log[UACR] (*Figure 3A*). Contrastingly, compared to participants in tertile 1 of hsCRP, participants in tertile 3 of hsCRP did not have significantly different annual rate of change in log[UACR] [β: 2.96 (95% CI: −0.74–6.66), *P* = 0.117], despite participants in tertile 2 having significantly greater annual increase in log[UACR] [*β* (in ×10^−3^ unit per year): 5.08 (95% CI: 1.56–8.61), *P* = 0.005] (*Figure 3B*). However, in the first sensitivity analysis, standardized log-transformed IL-6 was consistently associated with a significant annual increase in log[UACR] [*β* per SD-increment in log-transformed IL-6: 3.50 (95% CI: 2.17–4.83), *P* < 0.001] as was standardized log-transformed hsCRP [*β* per SD-increment in log-transformed hsCRP: 1.50 (95% CI: 0.26–2.73), *P* = 0.018]. In the second sensitivity analysis, further adjustments for the Agatston score yielded similar results as the main analyses (see Supplementary material online, *Table S4*).

Subgroup analyses for both IL-6 and hsCRP produced estimates that were directionally consistent in most subgroups (see Supplementary material online, *Table S4*). Both IL-6 and hsCRP showed significantly stronger associations in male participants (joint tests for subgroup interaction *P* = 0.015 and *P* = 0.028, respectively). For IL-6, significantly stronger associations were observed in participants aged ≥61 years old (joint test for subgroup interaction *P* = 0.039), and participants with more severe coronary calcification, regardless of the categorization of Agatston score (joint tests for subgroup interaction *P* = 0.006 (binarily categorized) and *P* < 0.001 (ordinally categorized)). No other statistically significant subgroup interactions were found.

### Incident chronic kidney disease

Amongst the 4564 participants included in the analysis of associations between IL-6 and incident CKD, 463 (10.1%) developed incident CKD and 395 (8.7%) died. Amongst the 4664 participants included in the analysis of associations between hsCRP and incident CKD, 478 (10.3%) developed incident CKD, and 407 (8.7%) died. Both cohorts had a median follow-up of 16.8 (interquartile range: 13.8–17.5) years. The unadjusted cause-specific 10-year incidence of CKD, stratified by tertiles of IL-6 and hsCRP, is summarized in Supplementary material online, *Table S5*.

**Figure 4:**
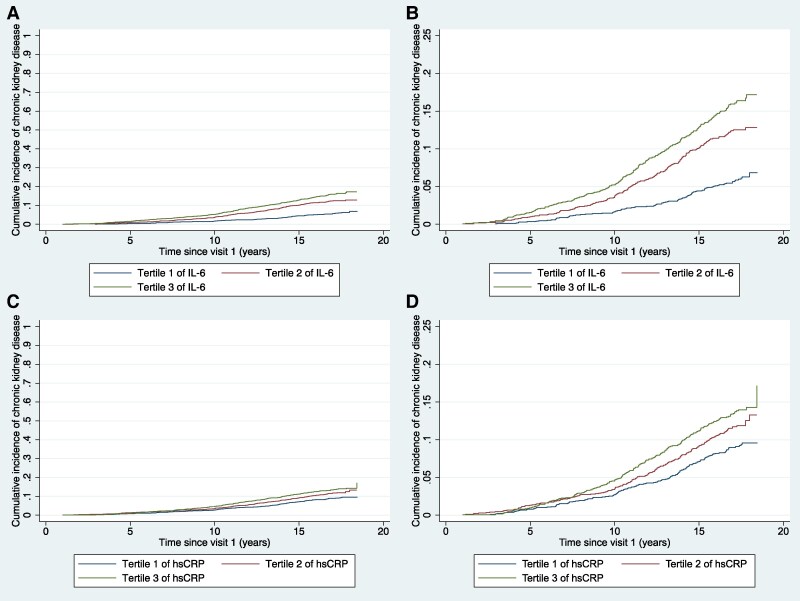
Aalen–johansen cumulative incidence curves of chronic kidney disease for each tertile of (*A*, *B*) interleukin-6 (IL-6) and (*C*, *D*) high-sensitivity C-reactive protein (hsCRP). Panels *B* and *D* present the same curves as Panels *A* and *B*, respectively, but with a magnified *y*-axis for easier Reading.

Results of the main and subgroup analyses for incident CKD are summarized in *Table 2*. Compared to participants in tertile 1 of IL-6, participants in tertile 2 [SHR: 1.43 (95% CI: 1.08–1.89), *P* = 0.013] and tertile 3 [SHR: 1.65 (95% CI: 1.23–2.20), *P* = 0.001] of IL-6 had significantly greater incidence of CKD (*Figure 4A* and *B*). In contrast, compared to participants in tertile 1 of hsCRP, neither participants in tertile 2 [SHR: 1.05 (95% CI: 0.82–1.34), *P* = 0.716] nor tertile 3 [SHR: 1.10 (95% CI: 0.85–1.44), *P* = 0.467] of hsCRP had significantly different incidence of CKD (*Figure 4C* and *D*). The first sensitivity analysis found that standardized log-transformed IL-6 was associated with the cumulative incidence of CKD [SHR per SD-increment in log-transformed IL-6: 1.20 (95% CI: 1.08–1.34), *P* = 0.001], while standardized log-transformed hsCRP showed no significant association [SHR per SD-increment in log-transformed hsCRP: 1.06 (95% CI: 0.95–1.19), *P* = 0.290]. Results of the second, third, and fourth sensitivity analyses are summarized in Supplementary material online, *Table S6*, all of which yielded similar results as the main analyses. Subgroup analyses for both IL-6 and hsCRP did not show any statistically significant interactions (see Supplementary material online, *Table S6*).

## Discussion

In this large multi-ethnic prospective cohort, we found that higher baseline IL-6 levels were independently associated with a faster rate of decline in eGFR, greater increases in albuminuria, and a higher risk of incident CKD over nearly two decades of follow-up. These relationships were robust across multiple models, sensitivity analyses, and most subgroups. These associations were stronger and more consistent for IL-6 than for hsCRP, which showed weaker or non-significant associations with albuminuria and incident CKD. Our findings reinforce the central role of inflammation in the pathogenesis of CKD within the CKM framework. In this paradigm, dysfunctional adipose tissue and cardiometabolic risk factors create a persistent inflammatory state that propagates subclinical vascular damage, atherosclerosis, and renal fibrosis.^1,25,26^ Elevated IL-6 likely reflects upstream immune activation that directly influences renal structures through pathways such as the NLRP3 inflammasome and JAK/STAT3 signalling.^27–33^ Animal models have shown that IL-6 can amplify glomerular injury, promote extracellular matrix deposition, and exacerbate proteinuria, particularly in the presence of hypertension or metabolic stress.^33^

Prior studies investigating inflammation and renal outcomes have reported mixed results, often limited by small sample sizes, homogeneous populations, or short follow-up.^34–38^ In the Beaver Dam Chronic Kidney Disease Study, Klein et al. found that higher inflammatory markers predicted incident CKD over 15 years,^12^ while the original MESA analysis by Hiramoto et al. linked markers such as hsCRP to 5-year declines in eGFR.^13^ Our study adds to this evidence by leveraging longer follow-up, a more diverse population, and robust competing risk models to examine the differential impact of upstream (IL-6) vs. downstream (hsCRP) markers. The stronger and more consistent associations seen for IL-6 suggest that measuring upstream cytokines may better capture the risk conferred by chronic low-grade inflammation. Notably, the magnitude of annual eGFR decline associated with higher IL-6 levels was modest in absolute terms but could become clinically significant when accumulated over many years. The associations were stronger among participants with higher baseline ASCVD risk, older age, and glycaemic dysregulation, supporting the idea that systemic metabolic and vascular stress amplify inflammation-mediated renal damage. This finding is also consistent with other MESA studies, which have demonstrated the association of inflammation with cardiovascular and metabolic outcomes, thereby reinforcing the broader CKM syndrome model, where interrelated pathways accelerate multiorgan injury.^1,6,26,39,40^

Our results have potential implications for risk stratification and targeted intervention. While hsCRP is widely used to gauge residual inflammatory risk in cardiovascular prevention, it may not fully capture upstream cytokine activity relevant to renal outcomes. Mendelian randomization studies suggest that genetically proxied IL-6 receptor blockade reduces coronary risk. The RESCUE (IL-6 inhibition with ziltivekimab in patients at high atherosclerotic risk) trial demonstrated that ziltivekimab—a novel IL-6 ligand inhibitor—lowers CRP by up to 92% in patients with CKD.^41,42^ Ongoing investigations, including the ZEUS trial, are evaluating whether IL-6 inhibition improves cardiovascular and renal outcomes in high-risk populations (CKD) with elevated inflammatory burden.^7,42^ If these trials demonstrate clinical benefit, IL-6 may emerge as both a therapeutic target and a biomarker for identifying patients who could benefit most from anti-inflammatory strategies. Pending further trial evidence, these data suggest that individuals with elevated IL-6 may warrant more vigilant monitoring of renal function to detect progressive decline and guide appropriate renoprotective strategies.

SGLT2 inhibitors confer cardio-renal benefits in part by improving endothelial function and suppressing vascular inflammation.^43–48^ They enhance nitric oxide bioavailability, reduce oxidative stress, and stabilize mitochondrial function in endothelial cells, promoting vasodilation and preserving vascular integrity. SGLT2 inhibitors downregulate pro-inflammatory pathways and cytokines such as interleukin-6, thereby limiting leukocyte recruitment, atherosclerotic progression, and microvascular injury in the heart and kidney. Through these endothelial and anti-inflammatory effects, in addition to their haemodynamic and metabolic actions, SGLT2 inhibitors lower albuminuria and slow the decline in glomerular filtration rate. Given the strong association between elevated IL-6 and progressive renal function decline among individuals with high atherosclerotic risk and dysglycaemia observed in this study, the use of SGLT2 inhibitors in this subset of patients is further substantiated as a therapeutic option to retard progressive renal function decline.

### Limitations

This study has several limitations that should be acknowledged. First, its observational design precludes any conclusions about causality. While we adjusted for a comprehensive set of confounders, the possibility of residual or unmeasured confounding remains. Second, IL-6 and hsCRP were each measured at a single baseline time point, which does not account for potential changes in inflammatory status over time. It is thus impossible to distinguish transient inflammation from persistent inflammation in this study, nor could the effects of longitudinal trends in inflammation be studied. Further studies exploring these may be informative. Third, the definition of incident CKD relied on clinical diagnoses captured through medical records and participant follow-up, which may be subject to misclassification or under-ascertainment. Fourth, although MESA’s diverse, multi-ethnic sample enhances generalizability, the cohort was drawn from relatively healthy, community-based adults without overt cardiovascular disease at baseline. As such, the rate of incident CKD was relatively low, and our findings may not fully extrapolate to higher-risk clinical populations, such as patients with advanced diabetes, established cardiovascular disease, or pre-existing CKD. Finally, the assays used for IL-6 measurement were standardized within MESA but are not routinely implemented in clinical practice, and assay variability or lack of standard thresholds could limit immediate translation to individual risk stratification.

Despite these limitations, the study’s large sample size, robust analytic methods, and long-term follow-up provide valuable evidence supporting the role of IL-6–mediated inflammation in progressive renal dysfunction.

## Conclusion

In this large, multi-ethnic prospective cohort, we demonstrated that higher baseline IL-6 levels were independently associated with greater long-term declines in eGFR, progressive worsening of albuminuria, and an increased risk of incident CKD over nearly two decades of follow-up. In contrast, hsCRP, a downstream marker of systemic inflammation, showed weaker and less consistent associations with these renal outcomes. These findings build on the growing body of evidence that inflammation, and IL-6 in particular, plays a central role in the interconnected pathways linking cardiovascular, metabolic, and kidney disease within the CKM framework. Taken together, our study provides robust, long-term epidemiologic evidence supporting the pivotal role of IL-6–mediated inflammation in progressive renal dysfunction. Future work should aim to validate these findings in other populations, investigate whether repeated IL-6 measurement adds predictive value beyond conventional risk factors, and determine whether interventions targeting upstream inflammatory pathways can translate into improved renal and cardiovascular outcomes.

## Supplementary Material

zwaf779_Supplementary_Data

## Data Availability

The data underlying this study are available through the National Heart, Lung, and Blood Institute Biologic Specimen and Data Repository Information Coordinating Center upon reasonable request.
