# Supplementary material for: The role of inflammation in longitudinal renal function decline and incident chronic kidney disease: the multi-ethnic study of atherosclerosis
Source: Eur J Prev Cardiol. 2025 Dec 23;33(5):718–28. doi: 10.1093/eurjpc/zwaf779 (PMC13021255; doi:10.1093/eurjpc/zwaf779)
Supplement: zwaf779_Supplementary_Data [file zwaf779_supplementary_data.docx]

**SUPPLEMENTARY MATERIAL**

**Supplementary Table 1.** Unadjusted estimated annual decline in estimated glomerular filtration rate, stratified by tertiles of interleukin-6 and high-sensitivity C-reactive protein. All estimates carry the unit of mL/min/1.73m^2^ per year. Estimates in brackets are 95% confidence intervals.

|  | Interleukin-6 | High-sensitivity C-reactive protein |
| --- | --- | --- |
| Tertile 1 (lowest) | 1.04 [0.98-1.10] | 1.11 [1.05-1.17] |
| Tertile 2 | 1.23 [1.16-1.30] | 1.20 [1.13-1.27] |
| Tertile 3 (highest) | 1.35 [1.30-1.45] | 1.33 [1.25-1.41] |

**Supplementary Table 2.** Summary of subgroup and sensitivity analyses results for longitudinal changes in estimated glomerular filtration rate (eGFR). All estimates represent differences in the annual eGFR reduction compared to tertile 1, with the unit of mL/min/1.73m^2^ per year. Bracketed numbers represent 95% confidence intervals. Only sensitivity analyses in which the exposure was analyzed in tertiles were summarized.

|  | | IL-6 | | | hsCRP | | |
| --- | --- | --- | --- | --- | --- | --- | --- |
|  |  | Tertile 1 | Tertile 2 | Tertile 3 | Tertile 1 | Tertile 2 | Tertile 3 |
| **Subgroup analyses** | | | | | | | |
| Age^1^ | ≤60 years old at visit 1  (IL-6: N=2205; hsCRP: N=2247) | 0 (reference) | 0.12 [-0.00-0.23], p=0.053 | 0.18 [0.05-0.21], p=0.007 | 0 (reference) | 0.00 [-0.11-0.12], p=0.960 | 0.14 [0.00-0.27], p=0.042 |
|  | ≥61 years old at visit 1  (IL-6: N=1938; hsCRP: N=1983) | 0 (reference) | 0.23 [0.09-0.38], p=0.001 | 0.45 [0.30-0.59], p<0.001 | 0 (reference) | 0.21 [0.07-0.34], p=0.003 | 0.27 [0.12-0.41], p<0.001 |
| ASCVD risk^2^ | <5% 10-year ASCVD risk (IL-6: N=2085; hsCRP: N=2115) | 0 (reference) | 0.03 [-0.09-0.14], p=0.650 | 0.14 [0.01-0.27], p=0.033 | 0 (reference) | -0.02 [-0.13-0.10], p=0.789 | 0.09 [-0.03-0.21], p=0.141 |
|  | ≥5% 10-year ASCVD risk (IL-6: N=1613; hsCRP: N=1662) | 0 (reference) | 0.26 [0.09-0.42], p=0.002 | 0.43 [0.27-0.60], p<0.001 | 0 (reference) | 0.17 [0.02-0.32], p=0.028 | 0.29 [0.12-0.46], p=0.001 |
| Baseline eGFR^3^ | Baseline eGFR <90 mL/min/1.73m^2^ (IL-6: N=3067; hsCRP: N=3125) | 0 (reference) | 0.22 [0.13-0.32], p<0.001 | 0.37 [0.26-0.47], p<0.001 | 0 (reference) | 0.09 [-0.01-0.19], p=0.072 | 0.16 [0.06-0.27], p=0.002 |
|  | Baseline eGFR ≥90 mL/min/1.73m^2^ (IL-6: N=1076; hsCRP: N=1105) | 0 (reference) | 0.08 [-0.12-0.28], p=0.423 | 0.19 [-0.01-0.39], p=0.062 | 0 (reference) | 0.07 [-0.12-0.26], p=0.495 | 0.17 [-0.03-0.38], p=0.095 |
| Glycemic dysfregulation^4^ | Without glycemic dysregulation (IL-6: N=3177; hsCRP: N=3237) | 0 (reference) | 0.15 [0.06-0.24], p=0.001 | 0.22 [0.12-0.32], p<0.001 | 0 (reference) | 0.10 [0.01-0.20], p=0.027 | 0.12 [0.02-0.22], p=0.018 |
|  | With glycemic dysregulation (IL-6: N=966; hsCRP: N=993) | 0 (reference) | 0.16 [-0.12-0.43], p=0.260 | 0.36 [0.10-0.62], p=0.007 | 0 (reference) | -0.04 [-0.29-0.21], p=0.772 | 0.19 [-0.07-0.44], p=0.149 |
| Sex^5^ | Male (IL-6: N=2194; hsCRP: N=2244) | 0 (reference) | 0.14 [0.03-0.26], p=0.016 | 0.21 [0.09-0.34], p=0.001 | 0 (reference) | 0.10 [-0.01-0.21], p=0.074 | 0.19 [0.05-0.33], p=0.009 |
|  | Female (IL-6: N=1949; hsCRP: N=1986) | 0 (reference) | 0.27 [0.14-0.39], p<0.001 | 0.39 [0.26-0.52], p<0.001 | 0 (reference) | 0.10 [-0.03-0.24], p=0.120 | 0.16 [0.03-0.29], p=0.013 |
| Hypertension^6^ | Without hypertension (IL-6: N=2526; hsCRP: N=2571) | 0 (reference) | 0.14 [0.04-0.24], p=0.006 | 0.17 [0.06-0.27], p=0.002 | 0 (reference) | 0.09 [-0.00-0.19], p=0.059 | 0.09 [-0.01-0.20], p=0.082 |
|  | With hypertension (IL-6: N=1617; hsCRP: N=1659) | 0 (reference) | 0.27 [0.11-0.44], p=0.001 | 0.41 [0.24-0.57], p<0.001 | 0 (reference) | 0.07 [-0.10-0.23], p=0.425 | 0.21 [0.05-0.37], p=0.012 |
| Obesity^7^ | Without obesity (IL-6: N=940; hsCRP: N=964) | 0 (reference) | 0.30 [0.15-0.45], p<0.001 | 0.22 [0.04-0.39], p=0.016 | 0 (reference) | 0.18 [0.03-0.33], p=0.016 | 0.25 [0.06-0.43], p=0.009 |
|  | With obesity (IL-6: N=3203; hsCRP: N=3266) | 0 (reference) | 0.15 [0.25-0.06], p=0.001 | 0.35 [0.25-0.44], p<0.001 | 0 (reference) | 0.04 [-0.05-0.14], p=0.394 | 0.19 [0.09-0.29], p<0.001 |
| BMI-defined obesity^8^ | Without BMI-defined obesity (IL-6: N=1063; hsCRP: N=1091) | 0 (reference) | 0.24 [0.10-0.38], p=0.001 | 0.25 [0.08-0.42], p=0.004 | 0 (reference) | 0.14 [0.00-0.27], p=0.047 | 0.23 [0.06-0.40], p=0.008 |
|  | With BMI-defined obesity (IL-6: N=3080; hsCRP: N=3139) | 0 (reference) | 0.16 [0.06-0.26], p=0.001 | 0.34 [0.24-0.45], p<0.001 | 0 (reference) | 0.05 [-0.05-0.14], p=0.344 | 0.19 [0.09-0.30], p<0.001 |
| Abdominal obesity^9^ | Without abdominal obesity (IL-6: N=1894; hsCRP: N=1934) | 0 (reference) | 0.21 [0.10-0.31], p<0.001 | 0.23 [0.12-0.35], p<0.001 | 0 (reference) | 0.17 [0.06-0.27], p=0.001 | 0.21 [0.08-0.34], p=0.002 |
|  | With abdominal obesity (IL-6: N=2249; hsCRP: N=2296) | 0 (reference) | 0.17 [0.05-0.29], p=0.007 | 0.37 [0.25-0.50], p<0.001 | 0 (reference) | -0.02 [-0.14-0.11], p=0.800 | 0.15 [0.02-0.28], p=0.023 |
| Ethnicity^10^ | White participants (IL-6: N=1812; hsCRP: N=1830) | 0 (reference) | 0.18 [0.06-0.30], p=0.003 | 0.32 [0.45-0.18], p<0.001 | 0 (reference) | 0.12 [0.00-0.24], p=0.041 | 0.26 [0.13-0.39], p<0.001 |
|  | Non-White participants (IL-6: N=2331; hsCRP: N=2400) | 0 (reference) | 0.22 [0.10-0.34], p<0.001 | 0.29 [0.17-0.41], p<0.001 | 0 (reference) | 0.09 [-0.03-0.20], p=0.147 | 0.13 [0.01-0.26], p=0.033 |
| Coronary calcification (binary)^11^ | With Agatston score of 0 (IL-6: N=2217; hsCRP: N=2262) | 0 (reference) | 0.16 [0.06-0.27], p=0.001 | 0.27 [0.17-0.38], p<0.001 | 0 (reference) | 0.14 [0.04-0.23], p=0.007 | 0.27 [0.16-0.38], p<0.001 |
|  | With Agatston score >0 (IL-6: N=1926; hsCRP: N=1968) | 0 (reference) | 0.25 [0.12-0.37], p<0.001 | 0.42 [0.29-0.55], p<0.001 | 0 (reference) | 0.08 [-0.05-0.20], p=0.233 | 0.22 [0.09-0.36], p=0.001 |
| Coronary calcification (ordinal)^12^ | With Agatston score of 0 (IL-6: N=2217; hsCRP: N=2262) | 0 (reference) | 0.16 [0.06-0.27], p=0.001 | 0.27 [0.17-0.38], p<0.001 | 0 (reference) | 0.14 [0.04-0.23], p=0.007 | 0.27 [0.16-0.38], p<0.001 |
|  | With Agatston score of >0 to <100 (IL-6: N=1055; hsCRP: N=1071) | 0 (reference) | 0.11 [-0.05-0.26], p=0.178 | 0.30 [0.14-0.47], p<0.001 | 0 (reference) | 0.01 [-0.15-0.17], p=0.923 | 0.14 [-0.03-0.30], p=0.103 |
|  | With Agatston score of ≥100 (IL-6: N=871; hsCRP: N=897) | 0 (reference) | 0.42 [0.22-0.63], p<0.001 | 0.55 [0.34-0.75], p<0.001 | 0 (reference) | 0.15 [-0.05-0.34], p=0.143 | 0.37 [0.15-0.60], p=0.001 |
| **Sensitivity analyses in which the exposure is analyzed in tertiles** | | | | | | | |
| Adjusting for log-transformed Agatston score | | 0 (reference) | 0.20 [0.13-0.28], p<0.001 | 0.36 [0.27-0.44], p<0.001 | 0 (reference) | 0.11 [0.03-0.19], p=0.005 | 0.25 [0.17-0.34], p<0.001 |
| Adjusting for Agatston score categories | | 0 (reference) | 0.20 [0.13-0.28], p<0.001 | 0.36 [0.27-0.44], p<0.001 | 0 (reference) | 0.11 [0.03-0.19], p=0.005 | 0.25 [0.17-0.34], p<0.001 |

^1^ Joint tests for age subgroup interactions: p=0.022 (IL-6); and p=0.061 (hsCRP)

^2^ Joint tests for ASCVD risk subgroup interactions: p=0.014 (IL-6); and p=0.065 (hsCRP)

^3^ Joint tests for baseline eGFR subgroup interactions: p=0.168 (IL-6); and p=0.959 (hsCRP)

^4^ Joint tests for glycemic dysregulation subgroup interactions: p=0.513 (IL-6); and p=0.281 (hsCRP)

^5^ Joint tests for sex subgroup interactions: p=0.112 (IL-6); and p=0.924 (hsCRP)

^6^ Joint tests for hypertension subgroup interactions: p=0.048 (IL-6); and p=0.320 (hsCRP)

^7^ Joint tests for obesity subgroup interactions: p=0.070 (IL-6); and p=0.196 (hsCRP)

^8^ Joint tests for BMI-defined obesity subgroup interactions: p=0.358 (IL-6); and p=0.440 (hsCRP)

^9^ Joint tests for abdominal obesity subgroup interactions: p=0.153 (IL-6); and p=0.090 (hsCRP)

^10^ Joint tests for ethnicity subgroup interactions: p=0.791 (IL-6); p=0.441 (hsCRP)

^11^ Joint tests for binarily categorized coronary calcification subgroup interactions: p=0.346 (IL-6); p=0.729 (hsCRP)

^12^ Joint tests for ordinally categorized coronary calcification subgroup interactions: p=0.130 (IL-6); p=0.518 (hsCRP)

BMI, body-mass index. eGFR, estimated glomerular filtration rate. hsCRP, high-sensitivity C-reactive protein. IL-6, interleukin-6.

**Supplementary Table 3.** Unadjusted estimated annual increase in logarithmically transformed urinary albumin-creatinine ratio, stratified by tertiles of interleukin-6 and high-sensitivity C-reactive protein. All estimates carry the unit of 10^-3^ unit per year. Estimates in brackets are 95% confidence intervals.

|  | Interleukin-6 | High-sensitivity C-reactive protein |
| --- | --- | --- |
| Tertile 1 (lowest) | 8.7 [6.4-10.9] | 10.1 [7.7-12.5] |
| Tertile 2 | 13.9 [11.2-16.6] | 14.9 [12.2-17.6] |
| Tertile 3 (highest) | 15.5 [12.5-18.6] | 13.1 [10.2-16.0] |

**Supplementary Table 4.** Summary of subgroup and sensitivity analyses results for longitudinal changes in log-transformed urinary albumin-creatinine ratio (log[UACR]). All estimates represent differences in the annual log[UACR] increase compared to tertile 1, with the unit of 10^-3^ unit per year. Bracketed numbers represent 95% confidence intervals. Only sensitivity analyses in which the exposure was analyzed in tertiles were summarized.

|  | | IL-6 | | | hsCRP | | |
| --- | --- | --- | --- | --- | --- | --- | --- |
|  |  | Tertile 1 | Tertile 2 | Tertile 3 | Tertile 1 | Tertile 2 | Tertile 3 |
| **Subgroup analyses** | | | | | | | |
| Age^1^ | ≤60 years old at visit 1  (IL-6: N=2241; hsCRP: N=2282) | 0 (reference) | 4.04 [-0.04-8.50], p=0.076 | 1.13 [-3.42-5.68], p=0.626 | 0 (reference) | 3.91 [-0.38-8.21], p=0.074 | 2.03 [-2.68-6.75], p=0.397 |
|  | ≥61 years old at visit 1  (IL-6: N=2026; hsCRP: N=2073) | 0 (reference) | 1.25 [-4.46-6.95], p=0.668 | 8.85 [2.73-14.97], p=0.005 | 0 (reference) | 5.23 [-0.56-11.01], p=0.077 | 3.85 [-2.08-9.77], p=0.203 |
| ASCVD risk^2^ | <5% 10-year ASCVD risk (IL-6: N=2115; hsCRP: N=2144) | 0 (reference) | 2.38 [-1.76-6.53], p=0.260 | -0.90 [-3.73-5.53], p=0.704 | 0 (reference) | 2.27 [-1.86-6.40], p=0.281 | 0.52 [-3.96-5.00], p=0.820 |
|  | ≥5% 10-year ASCVD risk (IL-6: N=1688; hsCRP: N=1739) | 0 (reference) | 1.44 [-5.58-8.45], p=0.688 | 8.41 [1.29-1.55], p=0.021 | 0 (reference) | 4.65 [-1.91-11.21], p=0.165 | 5.52 [-1.42-12.47], p=0.119 |
| Baseline eGFR^3^ | Baseline eGFR <90 mL/min/1.73m^2^ (IL-6: N=3164; hsCRP: N=3222) | 0 (reference) | 4.03 [0.00-8.06], p=0.050 | 9.23 [4.98-13.49], p<0.001 | 0 (reference) | 3.52 [-0.61-7.65], p=0.094 | 2.88 [-1.38-7.15], p=0.185 |
|  | Baseline eGFR ≥90 mL/min/1.73m^2^ (IL-6: N=1103; hsCRP: N=1133) | 0 (reference) | 3.76 [-3.18-10.70], p=0.288 | -0.48 [-8.02-7.06], p=0.900 | 0 (reference) | 9.99 [3.24-16.73], p=0.004 | 3.72 [-3.55-10.98], p=0.316 |
| Glycemic dysregulation^4^ | Without glycemic dysregulation (IL-6: N=3261; hsCRP: N=3321) | 0 (reference) | 4.36 [0.86-7.86], p=0.015 | 7.49 [3.59-11.40], p<0.001 | 0 (reference) | 4.50 [0.86-8.14], p=0.016 | 3.81 [0.01-7.60], p=0.049 |
|  | With glycemic dysregulation (IL-6: N=1006; hsCRP: N=1034) | 0 (reference) | -0.85 [-11.71-10.01], p=0.878 | 0.72 [-9.81-11.25], p=0.894 | 0 (reference) | 4.88 [-4.82-14.58], p=0.324 | -1.73 [-11.36-7.91], p=0.725 |
| Sex^5^ | Male (IL-6: N=2258; hsCRP: N=2309) | 0 (reference) | 4.73 [0.67-8.78], p=0.022 | 13.20 [8.75-17.65], p<0.001 | 0 (reference) | 4.49 [0.49-8.49], p=0.028 | 10.16 [5.54-14.78], p<0.001 |
|  | Female (IL-6: N=2009; hsCRP: N=2046) | 0 (reference) | 2.84 [-1.15-6.84], p=0.163 | 3.37 [-0.94-8.07], p=0.126 | 0 (reference) | 1.67 [-2.66-5.99], p=0.450 | 1.17 [-3.08-5.42], p=0.589 |
| Hypertension^6^ | Without hypertension (IL-6: N=2596; hsCRP: N=2640) | 0 (reference) | 3.29 [0.20-6.38], p=0.037 | 3.90 [0.30-7.51], p=0.034 | 0 (reference) | 2.63 [-0.49-5.76], p=0.098 | 3.46 [-0.02-6.94], p=0.051 |
|  | With hypertension (IL-6: N=1671; hsCRP: N=1715) | 0 (reference) | -0.56 [-6.70-5.58], p=0.858 | 6.50 [0.42-12.59], p=0.036 | 0 (reference) | -0.30 [-6.67-6.07], p=0.926 | -2.83 [-8.95-3.30], p=0.366 |
| Obesity^7^ | Without obesity (IL-6: N=967; hsCRP: N=990) | 0 (reference) | -0.19 [-5.56-5.17], p=0.944 | 6.44 [-0.65-13.54], p=0.075 | 0 (reference) | 2.06 [-3.68-7.79], p=0.482 | 2.24 [-3.82-8.30], p=0.468 |
|  | With obesity (IL-6: N=3300; hsCRP: N=3365) | 0 (reference) | 4.03 [0.59-7.47], p=0.022 | 7.69 [4.07-11.30], p<0.001 | 0 (reference) | 3.17 [-0.42-6.76], p=0.084 | 3.33 [-0.31-6.98], p=0.073 |
| BMI-defined obesity^8^ | Without BMI-defined obesity (IL-6: N=1094; hsCRP: N=1121) | 0 (reference) | 2.07 [-3.00-7.14], p=0.424 | 6.49 [-0.27-13.25], p=0.060 | 0 (reference) | 2.58 [-2.75-7.91], p=0.343 | 3.15 [2.57-8.88], p=0.280 |
|  | With BMI-defined obesity (IL-6: N=3173; hsCRP: N=3234) | 0 (reference) | 3.53 [-0.02-7.08], p=0.051 | 7.69 [3.99-11.39], p<0.001 | 0 (reference) | 3.13 [-0.55-6.82], p=0.096 | 3.33 [-0.40-7.05], p=0.080 |
| Abdominal obesity^9^ | Without abdominal obesity (IL-6: N=1954; hsCRP: N=1993) | 0 (reference) | 2.36 [-1.66-6.39], p=0.250 | 8.50 [3.53-13.48], p=0.001 | 0 (reference) | 3.72 [-0.33-7.78], p=0.072 | 5.55 [0.74-10.37], p=0.024 |
|  | With abdominal obesity (IL-6: N=2313; hsCRP: N=2362) | 0 (reference) | 3.88 [-0.42-8.18], p=0.077 | 7.03 [2.64-11.43], p=0.002 | 0 (reference) | 1.88 [-2.89-6.64], p=0.440 | 1.45 [-3.21-6.11], p=0.541 |
| Ethnicity^10^ | White participants (IL-6: N=1858; hsCRP: N=1877) | 0 (reference) | 1.98 [-1.97-5.94], p=0.326 | 9.68 [4.90-14.45], p<0.001 | 0 (reference) | 5.41 [1.11-9.70], p=0.014 | 5.37 [0.98-9.76], p=0.016 |
|  | Non-White participants (IL-6: N=2409; hsCRP: N=2478) | 0 (reference) | 4.13 [0.01-8.24], p=0.049 | 5.81 [1.62-10.01], p=0.007 | 0 (reference) | 1.00 [-3.11-5.11], p=0.633 | 1.36 [-2.86-5.58], p=0.528 |
| Coronary calcification (binary)^11^ | With Agatston score of 0 (IL-6: N=2260; hsCRP: N=2306) | 0 (reference) | 4.62 [0.91-8.32], p=0.015 | 2.81 [-1.18-6.80], p=0.168 | 0 (reference) | 1.76 [-1.97-5.49], p=0.355 | 1.79 [-2.22-5.80], p=0.381 |
|  | With Agatston score >0 (IL-6: N=2007; hsCRP: N=2049) | 0 (reference) | 1.09 [-3.41-5.60], p=0.634 | 10.51 [5.66-15.36], p<0.001 | 0 (reference) | 4.87 [0.16-9.59], p=0.043 | 5.67 [0.95-10.39], p=0.019 |
| Coronary calcification (ordinal)^12^ | With Agatston score of 0 (IL-6: N=2260; hsCRP: N=2306) | 0 (reference) | 4.62 [0.91-8.32], p=0.015 | 2.81 [-1.18-6.80], p=0.168 | 0 (reference) | 1.76 [-1.97-5.49], p=0.355 | 1.79 [-2.22-5.80], p=0.381 |
|  | With Agatston score of >0 to <100 (IL-6: N=1093; hsCRP: N=1109) | 0 (reference) | -2.90 [-8.65-2.56], p=0.324 | 2.08 [-3.89-8.04], p=0.495 | 0 (reference) | 4.99 [-0.94-10.92], p=0.099 | 2.86 [-2.82-8.54], p=0.323 |
|  | With Agatston score of ≥100 (IL-6: N=914; hsCRP: N=940) | 0 (reference) | 7.12 [-0.05-14.29], p=0.052 | 22.44 [14.54-30.35], p<0.001 | 0 (reference) | 4.63 [-3.02-12.28], p=0.236 | 10.93 [2.77-19.09], p=0.009 |
| **Sensitivity analyses in which the exposure is analyzed in tertiles** | | | | | | | |
| Adjusting for log-transformed Agatston score | | 0 (reference) | 3.36 [0.50-6.23], p=0.021 | 7.41 [4.29-10.53], p<0.001 | 0 (reference) | 5.09 [1.57-8.62], p=0.005 | 2.97 [-0.73-6.67], p=0.116 |
| Adjusting for Agatston score categories | | 0 (reference) | 3.36 [0.50-6.22], p=0.021 | 7.41 [4.29-10.53], p<0.001 | 0 (reference) | 5.09 [1.56-8.62], p=0.005 | 2.97 [-0.73-6.67], p=0.116 |

^1^ Joint tests for age subgroup interactions: p=0.039 (IL-6); and p=0.911 (hsCRP)

^2^ Joint tests for ASCVD risk subgroup interactions: p=0.140 (IL-6); and p=0.599 (hsCRP)

^3^ Joint tests for baseline eGFR subgroup interactions: p=0.073 (IL-6); and p=0.262 (hsCRP)

^4^ Joint tests for glycemic dysregulation subgroup interactions: p=0.374 (IL-6); and p=0.413 (hsCRP)

^5^ Joint tests for sex subgroup interactions: p=0.015 (IL-6); and p=0.028 (hsCRP)

^6^ Joint tests for hypertension subgroup interactions: p=0.213 (IL-6); and p=0.177 (hsCRP)

^7^ Joint tests for obesity subgroup interactions: p=0.645 (IL-6); and 0.917 (hsCRP)

^8^ Joint tests for BMI-defined obesity subgroup interactions: p=0.989 (IL-6); and 0.995 (hsCRP)

^9^ Joint tests for abdominal obesity subgroup interactions: p=0.712 (IL-6); and 0.473 (hsCRP)

^10^ Join tests for ethnicity subgroup interactions: p=0.178 (IL-6); p=0.227 (hsCRP)

^11^ Join tests for binarily categorized coronary calcification subgroup interactions: p=0.006 (IL-6); p=0.397 (hsCRP)

^12^ Join tests for ordinally categorized coronary calcification subgroup interactions: p<0.001 (IL-6); p=0.338 (hsCRP)

BMI, body-mass index. eGFR, estimated glomerular filtration rate. hsCRP, high-sensitivity C-reactive protein. IL-6, interleukin-6.

**Supplementary Table 5.** Unadjusted estimated 10-year incidence of chronic kidney disease, stratified by tertiles of interleukin-6 and high-sensitivity C-reactive protein. All estimates are percentages. Estimates in brackets are 95% confidence intervals.

|  | Interleukin-6 | High-sensitivity C-reactive protein |
| --- | --- | --- |
| Tertile 1 (lowest) | 1.6 [1.1-2.4] | 2.4 [1.7-3.4] |
| Tertile 2 | 3.6 [2.7-4.7] | 3.9 [2.9-5.0] |
| Tertile 3 (highest) | 5.6 [4.4-6.9] | 4.7 [3.7-5.9] |

**Supplementary Table 6.** Summary of subgroup and sensitivity analyses results for incident chronic kidney disease. All estimates are sub-hazard ratios. Bracketed numbers represent 95% confidence intervals. Only sensitivity analyses in which the exposure was analyzed in tertiles were summarized.

|  | | IL-6 | | | hsCRP | | |
| --- | --- | --- | --- | --- | --- | --- | --- |
|  |  | Tertile 1 | Tertile 2 | Tertile 3 | Tertile 1 | Tertile 2 | Tertile 3 |
| **Subgroup analyses** | | | | | | | |
| Age^1^ | ≤60 years old at visit 1  (IL-6: N=2371; CRP: N=2418) | 1 (reference) | 1.69 [1.0 4-2.72], p=0.033 | 2.26 [1.41-3.65], p=0.001 | 1 (reference) | 1.25 [0.77-2.03], p=0.367 | 1.36 [0.82-2.25], p=0.238 |
|  | ≥61 years old at visit 1  (IL-6: N=2193; CRP: N=2246) | 1 (reference) | 1.27 [0.89-1.81], p=0.193 | 1.39 [0.96-2.01], p=0.084 | 1 (reference) | 0.97 [0.72-1.31], p=0.858 | 1.01 [0.73-1.39], p=0.957 |
| ASCVD risk^2^ | <5% 10-year ASCVD risk (IL-6: N=2479; CRP: N=2515) | 1 (reference) | 1.56 [0.93-2.63], p=0.095 | 1.96 [1.16-3.31], p=0.012 | 1 (reference) | 1.12 [0.66-1.90], p=0.680 | 1.22 [0.71-2.08], p=0.468 |
|  | ≥5% 10-year ASCVD risk (IL-6: N=2085; CRP: N=2149) | 1 (reference) | 1.36 [0.93-1.97], p=0.110 | 1.54 [1.06-2.23], p=0.025 | 1 (reference) | 0.97 [0.71-1.32], p=0.837 | 1.14 [0.82-1.59], p=0.422 |
| Baseline eGFR^3^ | Baseline eGFR <90 mL/min/1.73m^2^ (IL-6: N=3371; CRP: N=3438) | 1 (reference) | 1.45 [1.06-1.98], p=0.021 | 1.65 [1.19-2.29], p=0.003 | 1 (reference) | 1.04 [0.79-1.37], p=0.764 | 1.08 [0.80-1.47], p=0.598 |
|  | Baseline eGFR ≥90 mL/min/1.73m^2^ (IL-6: N=1193; CRP: N=1226) | 1 (reference) | 1.41 [0.73-2.72], p=0.313 | 1.69 [0.88-3.25], p=0.113 | 1 (reference) | 1.20 [0.64-2.21], p=0.571 | 1.31 [0.73-2.36], p=0.370 |
| Glycemic dysregulation^4^ | Without glycemic dysregulation (IL-6: N=3465; CRP: N=3534) | 1 (reference) | 1.98 [1.14-3.42], p=0.015 | 2.35 [1.32-4.18], p=0.004 | 1 (reference) | 1.08 [0.78-1.40], p=0.646 | 0.99 [0.70-1.41], p=0.964 |
|  | With glycemic dysregulation (IL-6: N=1099; CRP: N=1130) | 1 (reference) | 1.24 [0.89-1.74], p=0.206 | 1.35 [0.94-1.92], p=0.102 | 1 (reference) | 1.04 [0.68-1.59], p=0.859 | 1.36 [0.87-2.13], p=0.175 |
| Sex^5^ | Male (IL-6: N=2412; hsCRP: N=2470) | 1 (reference) | 1.58 [1.13-2.21], p=0.008 | 1.62 [1.13-2.33], p=0.009 | 1 (reference) | 1.11 [0.83-1.50], p=0.487 | 1.15 [0.83-1.60], p=0.406 |
|  | Female (IL-6: N=2152; hsCRP: N=2194) | 1 (reference) | 1.15 [0.68-1.94], p=0.608 | 1.73 [1.02-2.93], p=0.041 | 1 (reference) | 0.88 [0.53-1.44], p=0.600 | 0.95 [0.59-1.52], p=0.845 |
| Hypertension^6^ | Without hypertension (IL-6: N=2739; hsCRP: N=2789) | 1 (reference) | 1.52 [0.99-2.34], p=0.053 | 1.68 [1.07-2.62], p=0.023 | 1 (reference) | 1.38 [0.90-2.11], p=0.143 | 1.25 [0.79-1.98], p=0.342 |
|  | With hypertension (IL-6: N=1825; hsCRP: N=1875) | 1 (reference) | 1.43 [0.98-2.09], p=0.066 | 1.68 [1.14-2.48], p=0.009 | 1 (reference) | 0.91 [0.67-1.24], p=0.552 | 1.04 [0.75-1.44], p=0.827 |
| Obesity^7^ | Without obesity (IL-6: N=1033; hsCRP: N=1059) | 1 (reference) | 1.20 [0.66-2.19], p=0.540 | 0.93 [0.43-2.02], p=0.860 | 1 (reference) | 1.27 [0.66-2.45], p=0.471 | 0.84 [0.37-1.91], p=0.684 |
|  | With obesity (IL-6: N=3531; hsCRP: N=3605) | 1 (reference) | 1.43 [1.04-1.97], p=0.027 | 1.74 [1.25-2.40], p=0.001 | 1 (reference) | 0.99 [0.75-1.30], p=0.926 | 1.09 [0.81-1.46], p=0.566 |
| BMI-defined obesity^8^ | Without BMI-defined obesity (IL-6: N=1170; hsCRP: N=1200) | 1 (reference) | 1.52 [0.87-2.67], p=0.142 | 1.01 [0.48-2.12], p=0.979 | 1 (reference) | 1.30 [0.71-2.40], p=0.398 | 1.07 [0.54-2.12], p=0.845 |
|  | With BMI-defined obesity (IL-6: N=3394; hsCRP: N=3464) | 1 (reference) | 1.36 [0.99-1.89], p=0.061 | 1.72 [1.24-2.39], p=0.001 | 1 (reference) | 0.97 [0.73-1.28], p=0.810 | 1.07 [0.80-1.44], p=0.647 |
| Abdominal obesity^9^ | Without abdominal obesity (IL-6: N=2076; hsCRP: N=2121) | 1 (reference) | 1.69 [1.13-2.53], p=0.011 | 1.40 [0.88-2.24], p=0.160 | 1 (reference) | 1.05 [0.70-1.57], p=0.800 | 1.22 [0.78-1.92], p=0.391 |
|  | With abdominal obesity (IL-6: N=2488; hsCRP: N=2543) | 1 (reference) | 1.33 [0.89-1.99], p=0.165 | 1.80 [1.19-2.72], p=0.006 | 1 (reference) | 1.02 [0.73-1.43], p=0.908 | 1.03 [0.74-1.43], p=0.875 |
| Ethnicity^10^ | White participants (IL-6: N=1949; hsCRP: N=1973) | 1 (reference) | 1.55 [1.01-2.38], p=0.044 | 1.73 [1.11-2.71], p=0.017 | 1 (reference) | 1.33 [0.91-1.96], p=0.142 | 1.07 [0.67-1.68], p=0.786 |
|  | Non-White participants (IL-6: N=2615; hsCRP: N=2691) | 1 (reference) | 1.35 [0.93-1.96], p=0.119 | 1.58 [1.08-2.31], p=0.020 | 1 (reference) | 0.87 [0.62-1.21], p=0.401 | 1.10 [0.78-1.53], p=0.592 |
| Coronary calcification (binary)^11^ | With Agatston score of 0 (IL-6: N=2394; hsCRP: N=2441) | 1 (reference) | 1.51 [0.98-2.34], p=0.061 | 1.69 [1.09-2.63], p=0.019 | 1 (reference) | 0.96 [0.61-1.50], p=0.862 | 1.40 [0.90-2.18], p=0.133 |
|  | With Agatston score >0 (IL-6: N=2170; hsCRP: N=2223) | 1 (reference) | 1.37 [0.94-2.00], p=0.096 | 1.65 [1.11-2.44], p=0.013 | 1 (reference) | 1.08 [0.79-1.47], p=0.645 | 0.98 [0.70-1.38], p=0.914 |
| Coronary calcification (ordinal)^12^ | With Agatston score of 0 (IL-6: N=2394; hsCRP: N=2441) | 1 (reference) | 1.51 [0.98-2.34], p=0.061 | 1.69 [1.09-2.63], p=0.019 | 1 (reference) | 0.96 [0.61-1.50], p=0.862 | 1.40 [0.90-2.18], p=0.133 |
|  | With Agatston score of >0 to <100 (IL-6: N=1180; hsCRP: N=1200) | 1 (reference) | 1.29 [0.73-2.29], p=0.380 | 2.02 [1.13-3.62], p=0.018 | 1 (reference) | 1.03 [0.62-1.70], p=0.914 | 0.99 [0.59-1.66], p=0.964 |
|  | With Agatston score of ≥100 (IL-6: N=990; hsCRP: N=1023) | 1 (reference) | 1.24 [0.73-2.12], p=0.422 | 1.33 [0.76-2.33], p=0.324 | 1 (reference) | 1.09 [0.71-1.67], p=0.681 | 1.01 [0.63-1.61], p=0.978 |
| **Sensitivity analyses in which the exposure is analyzed in tertiles** | | | | | | | |
| Adjusting for log-transformed Agatston score | | 1 (reference) | 1.42 [1.07-1.88], p=0.016 | 1.62 [1.21-2.17], p=0.001 | 1 (reference) | 1.05 [0.82-1.34], p=0.717 | 1.10 [0.84-1.43], p=0.491 |
| Adjusting for Agatston score categories | | 1 (reference) | 1.41 [1.06-1.87], p=0.017 | 1.62 [1.21-2.16], p=0.001 | 1 (reference) | 1.05 [0.82-1.34], p=0.717 | 1.09 [0.84-1.43], p=0.505 |
| Censoring incident CKD which occurred within three years of exam 1 (number of incident CKD cases censored: N=16 (IL-6) and N=17 (hsCRP)) | | 1 (reference) | 1.41 [1.07-1.88], p=0.016 | 1.60 [1.19-2.14], p=0.002 | 1 (reference) | 1.03 [0.80-1.33], p=0.816 | 1.11 [0.85-1.46], p=0.434 |
| Censoring incident CKD which occurred within five years of exam 1 (number of incident CKD cases censored: N=55 (IL-6) and N=60 (hsCRP)) | | 1 (reference) | 1.34 [1.00-1.80], p=0.048 | 1.50 [1.10-2.03], p=0.009 | 1 (reference) | 1.00 [0.77-1.30], p=0.988 | 1.07 [0.81-1.41], p=0.645 |
| Censoring at MACE which occurred within three years of exam 1 without prior incident CKD (number of cases censored: N=80 (IL-6) and N=83 (hsCRP)) | | 1 (reference) | 1.39 [1.05-1.86], p=0.023 | 1.59 [1.18-2.14], p=0.002 | 1 (reference) | 1.06 [0.82-1.37], p=0.666 | 1.12 [0.85-1.48], p=0.412 |

^1^ Joint tests for age subgroup interactions: p=0.541 (IL-6); and p=0.567 (CRP)

^2^ Joint tests for ASCVD risk subgroup interactions: p=0.590 (IL-6); and p=0.576 (CRP)

^3^ Joint tests for baseline eGFR subgroup interactions: p=0.908 (IL-6); and p=0.995 (CRP)

^4^ Joint tests for glycemic dysregulation subgroup interactions: p=0.263 (IL-6); and p=0.356 (CRP)

^5^ Joint tests for sex subgroup interactions: p=0.301 (IL-6); and p=0.444 (hsCRP)

^6^ Joint tests for hypertension subgroup interactions: p=0.873 (IL-6); and p=0.349 (hsCRP)

^7^ Joint tests for obesity subgroup interactions: p=0.398 (IL-6); and p=0.438 (hsCRP)

^8^ Joint tests for BMI-defined obesity subgroup interactions: p=0.135 (IL-6); and p=0.354 (hsCRP)

^9^ Joint tests for abdominal obesity subgroup interactions: p=0.151 (IL-6); and p=0.688 (hsCRP)

^10^ Join tests for ethnicity subgroup interactions: p=0.906 (IL-6); p=0.107 (hsCRP)

^11^ Join tests for binarily categorized coronary calcification subgroup interactions: p=0.717 (IL-6); p=0.143 (hsCRP)

^12^ Join tests for ordinally categorized coronary calcification subgroup interactions: p=0.698 (IL-6); p=0.437 (hsCRP)

BMI, body-mass index. eGFR, estimated glomerular filtration rate. hsCRP, high-sensitivity C-reactive protein. IL-6, interleukin-6. MACE, major adverse cardiovascular events.
